# From Rash Decisions to Critical Conditions: A Systematic Review of Dermatological Presentations in Emergency Departments

**DOI:** 10.3390/diagnostics15050614

**Published:** 2025-03-04

**Authors:** Abdullah S. Algarni, Safinaz M. Alshiakh, Sara M. Alghamdi, Mohammed A. Alahmadi, Abdulah W. Bokhari, Samar N. Aljubayri, Waad M. Almutairy, Najwa M. Alfahmi, Ramy Samargandi

**Affiliations:** 1 Department of Medicine, College of Medicine, University of Jeddah, Jeddah 23218, Saudi Arabia; 2 Department of Emergency Medicine, Faculty of Medicine, King Abdulaziz University, Jeddah 22252, Saudi Arabia; 3 Faculty of Medicine, Al-Baha University, Al-Baha 65779, Saudi Arabia; 4 College of Medicine, Taibah University, Medina 42353, Saudi Arabia; 5 College of Medicine, Umm AlQura University, Makkah 24381, Saudi Arabia; 6 College of Medicine, University of Jeddah, Jeddah 23218, Saudi Arabia; 7 Department of Surgery, College of Medicine, University of Jeddah, Jeddah 23218, Saudi Arabia

**Keywords:** dermatological emergencies, emergency, diagnostic accuracy, systemic complications, skin lesions, teledermatology, rash

## Abstract

**Background:** Dermatological emergencies are critical conditions requiring immediate attention due to their potential to escalate into life-threatening scenarios. Accurate diagnosis and timely management are essential to prevent severe complications, including systemic involvement and mortality. This systematic review summarizes findings on dermatological emergencies in emergency departments (EDs), focusing on diagnostic accuracy, hospitalization rates, systemic complications, and management strategies. **Methods:** A systematic literature review of studies on dermatological emergencies was conducted, encompassing 24 prospective and retrospective cohort studies, cross-sectional studies, and descriptive analyses. The review included diverse patient populations, examining dermatological presentations, diagnostic methods, treatment strategies, hospitalization rates, and adverse outcomes. Key outcome measures such as diagnostic accuracy, complications, mortality rates, and re-visit frequencies were analyzed. **Results:** The studies revealed high diagnostic accuracy, particularly in in-person evaluations, with teledermatology showing slightly lower but reliable rates. Systemic complications, including severe drug reactions, bacterial infections, and autoimmune diseases, were common causes of hospitalization. Mortality rates varied, with conditions such as toxic epidermal necrolysis showing the highest risk. Hospitalization rates averaged 4.52%, and re-visit rates ranged from 1% to 6.5%. The results also highlighted the impact of environmental factors and seasonal trends on dermatological presentations. **Conclusions:** Dermatological emergencies pose significant challenges in emergency care. High diagnostic accuracy and effective management strategies are crucial in preventing severe outcomes. Timely diagnosis, careful management of systemic complications, and teledermatology play critical roles in improving care. Future research should focus on standardized management protocols, telemedicine applications, and the influence of environmental and demographic factors to enhance patient outcomes.

## 1. Introduction

Dermatological conditions are a common reason for visits to emergency departments (EDs) worldwide [1,2]. While many skin presentations are benign and self-limiting, a subset of dermatological conditions can signify underlying systemic diseases or herald life-threatening emergencies [3,4]. The challenge for emergency care providers lies in distinguishing between benign and critical conditions, particularly when faced with subtle or atypical presentations [5].

Rashes are among the most frequent dermatological complaints encountered in EDs [1,6]. These presentations can range from innocuous allergic reactions to severe manifestations of infections, autoimmune diseases, or adverse drug reactions [7]. Misdiagnosis or delayed recognition of serious rashes may result in significant morbidity, prolonged hospital stays, or even mortality. For example, conditions like Stevens–Johnson syndrome (SJS), toxic epidermal necrolysis (TEN), and meningococcemia often begin with nonspecific rash patterns but rapidly progress to critical systemic involvement if not promptly identified and managed [8].

Accurate recognition of rash etiology requires a comprehensive understanding of clinical patterns, patient history, and the use of diagnostic tools [9]. The complexity of differential diagnosis for rashes is heightened in the ED due to time constraints, limited dermatological training among many emergency physicians, and the broad spectrum of potential causes, including infections, allergies, vasculitis, and systemic illnesses [10,11]. Moreover, the absence of specialized dermatological consultation services in many EDs compounds these challenges, leading to reliance on clinical judgment alone [11].

This systematic review aims to evaluate rash presentations in emergency settings, focusing on their recognition, accurate diagnosis, and timely management. By analyzing the underlying causes and outcomes of rash-related ED visits, this review seeks to provide insights into the causes of morbidity and the progression to critical conditions originating from dermatological emergencies.

## 2. Materials and Methods

### 2.1. Study Design and Population

The study was registered in the International Prospective Register of Systematic Reviews (PROSPERO) database statement (CRD42024623361). This systematic review focused on studies that assessed dermatological presentations in emergency departments (EDs), precisely skin rash conditions among all aged patients. Eligible studies included observational designs, case series with at least five participants, and case reports, all reporting on diagnostic and management strategies used in ED settings.

### 2.2. Search Strategy

This systematic review was conducted with strict adherence to the Preferred Reporting Items for Systematic Reviews and Meta-Analyses (PRISMA) guidelines [12]. A comprehensive literature search was conducted across multiple databases, including PubMed, Google Scholar, Web of Science, Wiley, EBSCO, and OVID. The literature search covered the period between January 2005 and December 2024. The search terms combined keywords related to dermatology, emergency care, and clinical presentations: (“Dermatological” OR “Dermatologic” OR “Dermatology”) AND (“Presentations” OR “Conditions” OR “Cases” OR “Consultations”) AND (“Emergency” OR “Emergencies” OR “ER” OR “ED”). Search terms were tailored for each database to ensure thorough retrieval of relevant studies. No restrictions were applied to publication dates, and only studies published in English were included.

### 2.3. Study Selection

Inclusion criteria encompassed observational studies, case series with a minimum of five participants, and case reports. Eligible populations included patients of all ages presenting to EDs with skin rash conditions. Studies focusing on chronic dermatological conditions unrelated to emergency care (e.g., psoriasis, chronic eczema) were excluded. Relevant outcomes included diagnostic accuracy, management efficacy, or morbidity reduction. Studies such as experimental research, systematic reviews, meta-analyses, expert opinion (e.g., editorials and letters), and non-English studies were excluded.

### 2.4. Screening and Data Management

All search results were imported into Mendeley reference management software (version 2.128.0) to facilitate the screening and selection process. Two researchers independently reviewed titles and abstracts to identify potentially eligible studies. Full-text articles were then assessed against inclusion and exclusion criteria. Any discrepancies in study selection were resolved through discussion or consultation with a third reviewer. The reference lists of included studies and relevant review articles were manually screened to identify additional studies that may have been missed during the database searches.

### 2.5. Data Extraction

Data were extracted using a standardized form, capturing study characteristics (design, location, year of publication, sample size, and study aim), patient demographics (age, gender distribution, comorbidities, rash characteristics, severity, and risk factors), diagnostic methods (clinical examination, laboratory tests, biopsies), management strategies (medications, procedures, specialist referrals, timeliness of care), and outcomes (symptom resolution, prevention of complications, length of ED or hospital stay).

### 2.6. Quality Assessment

The quality of the included studies was assessed using the Newcastle–Ottawa Quality Assessment Scale for case-control and cross-sectional studies [13]. The ROBINS-I tool was employed for nonrandomized comparative studies [14]. Studies with a high risk of bias or low methodological quality were excluded.

### 2.7. Data Synthesis and Analysis

Data were synthesized narratively, highlighting key findings related to diagnostic and management strategies, their effectiveness, and associated patient outcomes. Statistical analysis included effect size measures (e.g., mean differences, standardized mean differences) and confidence intervals where possible. Heterogeneity was assessed, and publication bias was evaluated using appropriate statistical tools.

## 3. Results

**Figure 1:**
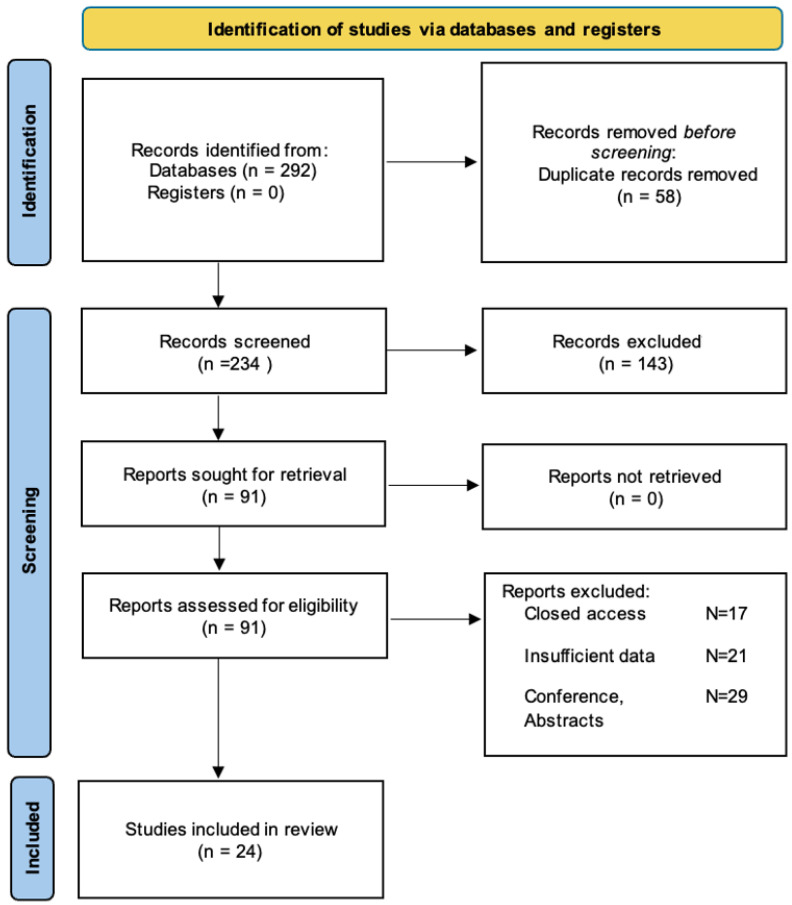
The PRISMA figures showing the steps to choose the studies for systematic review.

The reviewed 25 studies encompass a broad spectrum of methodologies, geographic regions, and sample sizes [1,6,15,16,17,18,19,20,21,22,23,24,25,26,27,28,29,30,31,32,33,34,35,36] (Figure 1). The research designs varied, including prospective and retrospective cohort studies, descriptive studies, cross-sectional analyses, and observational studies. Patient populations ranged from small cohorts of fewer than 300 participants to large datasets exceeding 50 million cases [24]. Key outcomes measured included diagnostic patterns, admission rates, hospitalization predictors, and dermatological emergencies’ prevalence. Multiple studies identified seasonal trends and the distribution of dermatological conditions, such as increased presentations during warmer months [15,19]. Other investigations focused on specific emergency conditions, diagnostic accuracy, or patient management approaches [27] (Table 1).

The age range of patients varied widely across studies, from infants as young as one month to elderly patients over 100 years old [18,22]. Female patients constituted a significant proportion of the study populations, typically representing 43–59% of cases [1,16,17]. Common dermatological presentations included maculopapular rashes, erythematous lesions, and systemic conditions such as angioedema or SJS [28]. Associated symptoms included mucosal involvement, itching, pain, and pruritus, with severity often influenced by comorbidities, age, and the presence of systemic involvement [25,33]. Several studies highlighted the influence of environmental and seasonal factors, with heat and humidity exacerbating dermatological emergencies [15,36] (Table 2).

Diagnostic and management strategies varied significantly, reflecting regional and institutional practices. Common diagnostic methods included ICD coding systems, clinical evaluations, and biopsies, with teledermatology emerging as a valuable adjunct in some settings [16,17,30]. Management approaches ranged from topical and systemic treatments to surgical interventions such as debridement and biopsies [31]. Hospitalization rates were generally low but varied depending on the severity of the conditions, with higher rates observed in cases of infectious dermatoses or drug-induced reactions [32,36]. Referral patterns also differed, with some studies reporting high rates of outpatient follow-up and dermatology consultations, while others highlighted the role of ED-based management [1,21]. Timeliness of care emerged as a critical factor in addressing severe dermatological emergencies, particularly during the COVID-19 pandemic, which influenced both patient presentations and treatment strategies [26,31] (Table 3).

The results reveal significant findings regarding the management and outcomes of dermatological emergencies in emergency departments. High diagnostic accuracy was reported, particularly in in-person evaluations, with teledermatology showing slightly lower but reliable rates [29,30]. Systemic complications, such as severe drug reactions, autoimmune diseases, bacterial infections, organ failure, and sepsis, were common causes of hospitalization, highlighting the potential for dermatological conditions to escalate into life-threatening scenarios [1,34]. Adverse effects, including superinfections and electrolyte imbalances, were prevalent in more complex cases, underscoring the importance of cautious therapeutic management [30,31]. Hospitalization rates across studies averaged 4.52%, reflecting the substantial burden on healthcare systems, while re-visit rates ranged from 1% to 6.5%, indicating a need for improved initial care and follow-up to enhance long-term outcomes [1,20] (Table 4).

### Risk of Bias Assessment

The studies reviewed showed variability in the risk of bias, influenced by study design, sample size, and methodology. Cohort and prospective studies (e.g., [15,26]) had lower risks of bias due to well-defined inclusion criteria and real-time data collection. Retrospective designs (e.g., [17,19]) were associated with moderate risks of selection and detection bias due to potential inaccuracies in historical records and missing data. Cross-sectional and observational studies, such as Kody (2020) and Shao et al. (2020), faced challenges in ensuring representativeness and controlling for confounders [24,33]. Overall, the studies with low risk of bias provide robust evidence for the topic, while those with moderate to high risk should be interpreted cautiously, especially regarding outcomes like admission rates and systemic complications (Table 5).

## 4. Discussion

The findings from the reviewed studies underscore the critical importance of accurate diagnosis and timely management in dermatological emergencies. The significant variation in study designs, ranging from cohort studies to cross-sectional analyses, highlights the diverse approaches to assessing dermatological emergencies, but they collectively demonstrate consistent patterns in the prevalence, presentation, and management of these conditions. Seasonal trends, with increased presentations during warmer months, are consistent across several studies [15,19,36], and they emphasize the impact of environmental factors such as heat and humidity on dermatological conditions [37,38,39]. This suggests that dermatological conditions may be exacerbated or more frequently encountered in certain seasons, necessitating targeted public health strategies to address these seasonal fluctuations [40,41]. Moreover, comorbidities and age were shown to play significant roles in the severity of dermatological presentations, particularly in older patients or those with underlying health conditions [25,33]. These findings suggest that a comprehensive understanding of the patient’s overall health status is critical in predicting and managing the severity of dermatological emergencies.

The variation in diagnostic and management approaches across regions and institutions also stands out. The use of teledermatology emerged as a promising tool, particularly in resource-limited settings, although its diagnostic accuracy was slightly lower than in-person evaluations [29,30]. This finding aligns with previous research highlighting the growing role of telemedicine in enhancing access to dermatological care, particularly during the COVID-19 pandemic, which significantly impacted patient care and healthcare delivery [42,43]. The evidence suggests that teledermatology could be a valuable adjunct in improving early diagnosis and management, although in-person evaluations remain the gold standard in complex cases where a thorough physical examination is necessary [44].

Systemic complications and the need for hospitalization were another key outcome of the studies reviewed. The identification of severe drug reactions, infections, and autoimmune diseases as significant contributors to hospitalization supports the notion that dermatological emergencies often have systemic implications that require urgent and intensive care [1,34]. While the overall hospitalization rate across studies was relatively low (4.52%), the high severity of certain cases, particularly those involving infectious or drug-induced dermatoses, highlights the need for specialized care and comprehensive management strategies [32,36]. Furthermore, the findings suggest that early identification and timely management can significantly reduce the risk of severe complications and fatalities, which is consistent with previous studies that emphasize the importance of rapid diagnosis and intervention in dermatological emergencies [45].

Mucosal involvement plays a crucial role in evaluating the severity of dermatological emergencies, particularly in cases where exanthems are present. The presence and pattern of enanthems associated with exanthems can serve as key clinical clues in predicting disease progression. Enanthems may precede or accompany exanthems and are frequently observed in infectious and drug-induced dermatological conditions. Conditions such as measles and varicella often present with enanthems, whereas drug-induced reactions like SJS and TEN exhibit extensive mucosal erosions [46]. Although not all studies included in this systematic review provided detailed descriptions of oropharyngeal lesions, recognizing mucosal involvement remains essential for clinicians in emergency settings to refine their differential diagnoses and improve patient outcomes.

Adverse effects, including superinfections and electrolyte imbalances, were also a significant concern, particularly in complex cases. The prevalence of these complications underscores the need for cautious therapeutic management, particularly in patients with underlying health conditions or those receiving systemic treatments [30,31]. The importance of personalized treatment plans that take into account both the dermatological condition and the patient’s broader health profile is clear. Additionally, the wide variation in re-visit rates across studies, ranging from 1% to 6.5%, suggests that there may be gaps in the initial management or follow-up care that could be addressed to improve patient outcomes [1,20]. This finding highlights the need for more robust post-discharge follow-up strategies and better communication between emergency departments and outpatient care providers. 

Studies indicate dermatological complaints constitute approximately 4% to 8% of all emergency department (ED) visits [6]. However, only a small fraction of these consultations are for life-threatening conditions. A study in India found that 21% of 100 emergency dermatology consultations were deemed true dermatologic emergencies [34]. Similarly, a study in Turkey reported that 24.7% of dermatology consultations were classified as true dermatologic emergencies [23]. These variations highlight how regional and healthcare setting differences influence the prevalence of life-threatening dermatologic conditions in the ED.

The study has several limitations, including variability and heterogeneity among the included studies, encompassing different designs, populations, and geographic regions. This diversity introduces inconsistencies in diagnostic and management practices, as well as in outcome measures like hospitalization and revisit rates. The reliance on retrospective data increases the risk of bias due to incomplete or inaccurate records, while variability in diagnostic methods and differences in healthcare settings further challenge result comparability. Additionally, data collected during the COVID-19 pandemic, when healthcare delivery was atypical, may skew findings related to patient presentations and care timeliness. Future research should adopt standardized protocols and prospective studies to reduce variability and improve the robustness of conclusions.

## 5. Conclusions

While the studies reviewed offer valuable insights into the management and outcomes of dermatological emergencies, they also underscore the complexity of these conditions and the need for ongoing research to refine diagnostic and treatment strategies. Future research should aim to explore more standardized management protocols, the role of telemedicine in dermatology, and the long-term outcomes of patients presenting with severe dermatological emergencies. Additionally, a more nuanced understanding of the impact of environmental factors, age, and comorbidities on dermatological conditions could further inform clinical practice and public health strategies to better address dermatological emergencies across diverse patient populations.

## Figures and Tables

**Table 1:** General characteristics of the studies.

Authors (Year)	Country of Origin	Study Design	Total Number of Patients Included	Outcomes Being Measured
Drago [15] (2014)	Italy	Prospective Cohort Study	372	Symptoms/signs of dermatological emergencies
Lai-Kwon [16] (2014)	Australia	Cohort study	4817	Classification of different dermatological presentations
Lai-Kwon [17] (2014)	Australia	Retrospective cohort study	4817	Dermatological presentations and admission rates
Ansorge [18] (2018)	Germany	Prospective single-center survey	1552	Demographics, diagnoses, hospitalization rates
Rubegni et al. [19] (2014)	Italy	Retrospective cohort study	12,226	Consultations, hospitalization, diagnostic groups
Martínez-Martínez et al. [20] (2010)	Spain	Retrospective, descriptive study	3662	Skin complaints in ED
Grillo [21] (2013)	Spain	Prospective cohort study	861	Emergency visit justification
Bancalari-Díaz [22] (2016)	Spain	Descriptive study	3084	Frequency of dermatologic emergencies
Özkur [23] (2020)	Turkey	Retrospective cohort study	444	Diagnoses and treatment outcomes
Kody [24] (2020)	USA	Cross-sectional study	51,809,000	Trends and types of ED visits
Kedia [25] (2023)	India	Observational study	202	Severity of emergencies
Temel [26] (2023)	Turkey	Cohort study	639	Consultations and disease profiles
Wang [27] (2009)	Singapore	Retrospective descriptive study	401	Conditions, demographics, admission rates
Bin Rubaian et al. [28] (2024)	Saudi Arabia	Retrospective chart review study	301	Discharge status, treatment, seasonal distribution
Kilic et al. [1] (2019)	Turkey	Retrospective cross-sectional study	859	Diagnoses, consultation, admission rates
Wallett & Sidhu. [35] (2012)	Australia	observational study	1283	Diagnosis, management pathways, admission rates
Jack et al. [34] (2011)	United States	Retrospective chart review	204	Conditions, admissions, prevalence
Alshibani et al. [6] (2024)	Saudi Arabia	Retrospective cohort study	11,443	Incidence, diagnoses, demographics
Abedini [29] (2017)	Iran	Retrospective observational study	2539	Emergencies, hospitalization, referral patterns
Hines et al. [30] (2021)	United States	Retrospective chart review	450 consultations (covering 438 patients)	Diagnostics, admissions, teledermatology differences
Isoletta et al. [31] (2020)	Italy	Retrospective observational study	197	ED visits during COVID-19
Pelloni et al. [32] (2019)	Switzerland	Prospective cross-sectional study	2390	Diagnostics, hospitalizations, follow-ups
Shao et al. [33] (2020)	Australia	Retrospective study with a prospective survey component	11,861	Common conditions, admissions, low-acuity visits
El Arabi et al. [36] (2022)	Morocco	Retrospective case series	843	Prevalence, profiles, hospitalization

**Table 2:** Patient’s characteristics.

Authors	Mean Age in Years (Range)	Female (%)	Rash Onset	Distribution/Morphology	Associated Symptoms	Most Prevalent Diagnosis	Risk Factors
Drago [15] (2014)	NR (11–90)	53.0	-	-	-	-infection (41.6%) -atypical exanthem (13.9%) -vasculitis (11.2%)	Summer, heat, humidity
Lai-Kwon [16] (2014)	44.2 (NR)	43.0	-	-	-	-cellulitis (36.1%) -allergy and skin involvement (19.5%) -boils/furuncles/pilonidal sinuses (11.1%)	-
Lai-Kwon [17] (2014)	49.2 (18–92)	59.0	-	-	-	-cellulitis (56.6%) -boils/furuncles/pilonidal sinuses (19%) -non-specific skin (3%)	Social disadvantage, homelessness
Ansorge [18] (2018)	41 (0–92)	53.5	>1 week	Generalized or extremities/head/neck	Itching, rash, pain	-eczema (9.7%), -urticaria (7.6%), -scabies (5.6%)	-
Rubegni et al. [19] (2014)	NR (0 to >65)	54.0	-	-	-	-Infections (27.1%), -non-specific and descriptive diagnosis (22.5%) -skin conditions caused by mechanical/physical agents (13.1%)	Seasonal factors (summer)
Martínez-Martínez et al. [20] (2010)	27.7 (1 months–96 years)	NA	-	-	27 Cases reported oral pathology (stomatitis (13), mouth ulcers (7), Mucositis (4), Glossitis (3))	-infectious diseases (47.49%) -urticaria and angioedema (20.13%) -non-specific (11.93%)	-
Grillo [21] (2013)	47, 2 (months–97 years)	58.3	-	-	Itching, pain, worry, 21 Cases reported Mucosal disorders (traumatic ulcer (9), mouth ulcers (8))	-inflammatory conditions (61.7%) -infections (32.9%) -tumors (4.1%)	-
Bancalari-Díaz [22] (2016)	44, (1 month–101 years)	54.1	-	-	43 cases reported (Aphthous ulcers (16), others (27))	-infectious skin diseases (23%) -eczema and dermatitis (15.1%) -surgical procedures and complications thereof (9.5%)	-
Özkur [23] (2020)	44.6 (18 to >65)	43.5	-	-	-	-infections (86.9%) -inflammatory dermatoses (5.4%) -urticaria and angioedema (5.1%)	-
Kody [24] (2020)	NR	NR	-	-	-	-cellulitis (1.2–1.3%) -cutaneous abscess (0.8–0.9%)	-
Kedia [25] (2023)	42.85 (5 months–96 years)	51.0	Acute/exacerbation	Localized/generalized.	Pain, pruritus, vesicles, ulcers, Mucosal involvement in 84 cases reported (Outpatient (36), Inpatient (48))	-acute urticaria (24.24%) -cutaneous adverse drug reactions (23.27%) -vesiculobullous diseases (10.89%)	Comorbidities, systemic involvement
Temel [26] (2023)	44.4 (SD ± 18.6)	48.8	-	-	-	Pre-pandemic period: -herpes zoster (20.8%) -urticaria (11.3%) -allergic contact dermatitis (8.4%) Pandemic period: -herpes zoster (29.1%) -other dermatitis (13.4%) -urticaria (9.3%)	-
Wang [27] (2009)	37.9 (1–106)	34.7	-	-	-	-chickenpox and herpes zoster (20.8%) -dermatitis/eczema (11.6%) -urticaria (11.4%)	-
Bin Rubaian et al. [28] (2024)	12 (4–30)	56.0	Not specified	-	Viral infections, eczema, maculopapular rash	-maculopapular rashes (35.55%) -viral infections (22.26%) -eczema (19.94%)	Seasonal/demographic trends
Kilic et al. [1] (2019)	39 (18–89)	59.5	Not specified	-	Itching, erythema, breathing difficulty, erythematous rash, pruritus	-urticaria with drug eruptions (84.5%) -angioedema and anaphylaxis (14.4%) - pruritic urticarial papules and plaques of pregnancy (0.7%)	-
Wallett & Sidhu. [35] (2012)	NA (18–29)	44.4	-	-	Common infections, ulcers, Cellulitis, abscess, eczema	-cellulitis (25.6%) -urticaria (14.4%) -abscess (9.5%)	-
Jack et al. [34] (2011)	43 (18–92)	38.0	Acute (<1 month)	Generalized/localized	Pain, pruritus, blistering, 32 cases with mucosal involvement “Moreover, the presence of blistering, erosive, or mucosal lesions is significantly more likely to be associated with an emergency diagnosis”	-eczematous dermatitis not otherwise specified (8.9%) -scabies (7.2%) -contact dermatitis (6.6%)	-
Alshibani et al. [6] (2024)	22.4 (1 month–103 years)	45.1	-	-	Rash, cellulitis, urticaria, burns, 1322 cases reported “Viral infections characterized by skin and mucous membrane lesions”	-rash and non-specific skin eruptions (16%) -cellulitis (13.6%) -urticaria (12.2%)	-
Abedini [29] (2017)	31.16 (3 days–92 years)	30.6	Acute (<5 days)	-	Pruritus, blistering, Shingles, insect bites, scabies	-infection and infestation (41.9%) -urticaria (16.7%) -dermatitis (13.2%)	Age, seasonal variation
Hines et al. [30] (2021)	41 (13–64)	56.0	Acute worsening	-	Dermatitis, infections, drug reactions	-dermatitis (24.7%) -infection (20.4%) -drug reaction (10.3%)	-
Isoletta et al. [31] (2020)	44.7 (SD ± 23.7)	45.6	Not reported	-	Eczema, infections, urticaria	-urticarial rashes (21.2%) -acute eczema (15.2%) -infectious diseases (15.2%)	Pandemic factors
Pelloni et al. [32] (2019)	44.9 (2 months–98 years)	55.2	Within 3 days (34.7%)	-	Infectious, eczema, urticaria	-infectious diseases (32.8%) -eczema (24.8%)	Older age
Shao et al. [33] (2020)	47.1 (SD ± 19.51)	41.5	-	-	Cellulitis, abscess, ulcers	-cellulitis (25%) -abscess (19%) -rash (17%)	Older age, male sex, infections
El Arabi et al. [36] (2022)	46.95 (SD ± 15.69)	46.8	Acute (<5 days)	-	Infectious, drug-induced, inflammatory	-infectious dermatoses (55.63%) -drug-Induced skin reactions (18.98%) -inflammatory dermatoses (13.4%)	Poor hygiene, diabetes, limited healthcare

SD: standard deviation, NR: not reported.

**Table 3:** Intervention Characteristics.

Author(s)	Diagnostic Methods	Management Strategies	Procedures Performed	Referral to Specialists	Timeliness of Care
Drago [15]	NR	NR	NR	NR	NR
Lai-Kwon [16]	Tele-dermatology and multimedia messaging systems	NR	NR	NR	NR
Lai-Kwon [17]	ICD-10 codes, clinical assessments	Admission to appropriate units, dermatological consultations	NR	Dermatology consultations as needed	Median time to doctor: 27 min
Ansorge [18]	Questionnaires, clinical diagnosis, medical records	Prior treatment common; 8.1% required hospitalization	NR	71.5% self-referred; 31.2% saw EDU same day	Not specified
Rubegni et al. [19]	Clinical evaluation, computerized records	NR	NR	NR	NR
Martínez-Martínez et al. [20]	Clinical diagnosis by nonspecialist physicians	NR	NR	NR	NR
Grillo [21]	Clinical evaluation, diagnostic tests	Direct discharge or referrals as needed	Diagnostic tests, skin biopsies	30.4% referred to outpatient dermatology	NR
Bancalari-Díaz [22]	Clinical evaluations by residents	Hospitalization, follow-up visits	290 surgical procedures (biopsies, sutures)	NR	NR
Özkur [23]	Electronic medical records	Antivirals, antihistamines, corticosteroids	NR	110 patients were true dermatological emergencies	NR
Kody [24]	National Hospital Ambulatory Survey	Education on managing cutaneous infections	NR	Not specified	Not specified
Kedia [25]	Clinical examination	Urgent interventions based on grading	NR	NR	NR
Temel [26]	ICD-10 codes, consultation records	NR	NR	NR	Consultation response time: 44.4 min (pre-pandemic); 60.3 min (pandemic)
Wang [27]	ICD-9 codes, consultation notes	Treatment and discharge protocols	Nail avulsions	31.3% referred to secondary care	NR
Bin Rubaian et al. [28]	ICD-10 codes, CTAS classifications	Topical treatments (steroids/antihistamines: 32%), systemic treatments (antibiotics, antivirals, etc.)	Primarily medical care	Primarily medical care	Median visit duration: 312 min; longer for admitted cases (333 min vs. 248 min)
Kilic et al. [1]	ICD-10 coding	Specialist consultations, prescriptions	NR	6.4% received consultations	NR
Wallett & Sidhu [35]	Clinical evaluations by ED/dermatology registrars	92.2% discharged; some outpatient referrals; 11.4% dermatology input	NR	64 outpatient referrals (92.3% attendance)	Cases categorized as non-urgent, semi-urgent, or urgent
Jack et al. [34]	Clinical evaluation, biopsies	Admission for severe conditions (e.g., SJS, pemphigus); outpatient care for others	Biopsies for complex cases	NR	NR
Alshibani et al. [6]	ICD-10 codes, clinical review	ED or outpatient management, teledermatology	Burn care, infection management	Dermatology consultations for complex cases	NR
Abedini [29]	Standard evaluations, differential diagnoses	Hospitalization (2.6%) or outpatient management	NR	1.37% referred by other physicians	NR
Hines et al. [30]	In-person and teledermatology consultations	Recommendations provided in 90% of cases	Biopsies (9.6%)	NR	Real-time consultations; results communicated before ED discharge
Isoletta et al. [31]	Clinical evaluations, biopsies	Increased topical (82.9–90.9%) and systemic (67.7–84.8%) treatments	Biopsy usage rose from 3.7% to 15.1%	NR	Delayed presentations noted in 2020
Pelloni et al. [32]	Clinical diagnosis	Hospitalization for severe cases (cellulitis, herpes zoster, drug reactions)	7.7% hospitalization	21.9% referred by primary care physicians	NR
Shao et al. [33]	Medical records, keyword searches	35% hospitalized for dermatology-related conditions	IV antibiotics, incision and drainage	Most referred by HCPs	NR
El Arabi et al. [36]	Clinical evaluations, imaging	Ambulatory care for most; hospitalization for severe conditions	NR	27.4% required specialist consultations	NR

NR: Not reported, HCPs: healthcare professionals, SJS: Stevens–Johnson syndrome.

**Table 4:** Outcome Measures.

Outcome	Main Findings
Numbers of Patients Diagnosed Accurately	- Abedini: 94.3% of patients diagnosed accurately at the first visit.
	- Hines: Concordance rates: In-person (93.5% primary, 93.7% aggregate), Teledermatology (88.2% primary, 87.2% aggregate).
Number of Patients with Systemic Complications	- Kilic: 19 patients hospitalized for systemic complications.
	- Jack: Severe complications like drug eruptions and autoimmune diseases.
	- Alshibani: Severe infections and burns managed.
	- Hines: Severe drug reactions and infections noted.
	- Pelloni: Includes bacterial infections and drug-induced reactions.
	- Shao: Infectious-related complications, e.g., cellulitis.
Mortality (Number of Deaths)	- Drago: Reported 3 deaths.
	- Kedia: Mortality rate of 1.98% (4 deaths).
	- Bin Rubaian: No deaths reported.
Type and Frequency of Adverse Effects	- Jack: Severe drug reactions as significant findings.
	- Hines: Severe cutaneous adverse reactions (6.9%) noted.
	- Isoletta: Higher complexity cases in 2020.
	- El Arabi: Drug-induced reactions noted.
Total Number of Hospitalized Patients	Across the studies, 2956 patients were hospitalized, with an overall hospitalization rate of 4.52%.
Total Number of Re-visits Within 7 Days	- Martínez-Martínez: Re-visit rate of 3.41%.
	- Grillo: Re-visit rate of 1%.
	- Bin Rubaian: Re-visit rate of 3.32%.
	- Kilic: Re-visit rate of 6.5%.

**Table 5:** Risk of Bias Table.

Study	Study Design	Selection Bias	Performance Bias	Detection Bias	Attrition Bias	Reporting Bias	Overall Risk of Bias
Drago [15]	Prospective cohort	Low	Low	Low	Low	Low	Low
Lai-Kwon [16]	Cohort study	Moderate	Low	Moderate	Low	Low	Moderate
Lai-Kwon [17]	Retrospective cohort	Moderate	Low	Moderate	Low	Low	Moderate
Ansorge [18]	Prospective survey	Low	Low	Low	Low	Low	Low
Rubegni et al. [19]	Retrospective cohort	Moderate	Low	Moderate	Low	Low	Moderate
Martínez-Martínez et al. [20]	Retrospective descriptive	Moderate	Low	High	Low	Moderate	Moderate
Grillo [21]	Prospective cohort	Low	Low	Low	Low	Low	Low
Bancalari-Díaz [22]	Descriptive	Moderate	Low	Moderate	Moderate	Moderate	Moderate
Özkur [23]	Retrospective cohort	Moderate	Low	Moderate	Low	Moderate	Moderate
Kody [24]	Cross-sectional	High	Low	High	Low	High	High
Kedia [25]	Observational	Moderate	Low	Moderate	Low	Moderate	Moderate
Temel [26]	Cohort study	Low	Low	Low	Low	Low	Low
Wang [27]	Retrospective descriptive	Moderate	Low	Moderate	Low	Moderate	Moderate
Bin Rubaian et al. [28]	Retrospective chart review	Moderate	Low	Moderate	Low	Low	Moderate
Kilic et al. [1]	Retrospective cross-sectional	Moderate	Low	Moderate	Low	Moderate	Moderate
Wallett & Sidhu. [35]	Observational	Moderate	Low	Moderate	Low	Moderate	Moderate
Jack et al. [34]	Retrospective chart review	Moderate	Low	Moderate	Low	Moderate	Moderate
Alshibani et al. [6]	Retrospective cohort	Moderate	Low	Moderate	Low	Low	Moderate
Abedini [29]	Retrospective observational	Moderate	Low	Moderate	Low	Low	Moderate
Hines et al. [30]	Telemedicine evaluation study	Low	Low	Low	Low	Low	Low
Isoletta et al. [31]	Retrospective observational	Moderate	Low	Moderate	Low	Moderate	Moderate
Pelloni et al. [32]	Prospective cross-sectional	Low	Low	Low	Low	Low	Low
Shao et al. [33]	Retrospective with survey	Moderate	Low	Moderate	Moderate	Moderate	Moderate
El Arabi et al. [36]	Retrospective case series	Moderate	Low	Moderate	Low	Moderate	Moderate

## Data Availability

The data presented in this study are available upon request from the corresponding author.
